# Shape of ligand immobilized particles dominates and amplifies the macrophage cytokine response to ligands

**DOI:** 10.1371/journal.pone.0217022

**Published:** 2019-05-17

**Authors:** Anusha Garapaty, Julie A. Champion

**Affiliations:** School of Chemical & Biomolecular Engineering, Georgia Institute of Technology, Atlanta, Georgia, United States of America; LAAS-CNRS, FRANCE

## Abstract

Macrophages aid in clearing synthetic particulates introduced into the body and bridge innate and adaptive immunity through orchestrated secretion of cytokines and chemokines. While the field has made tremendous progress in understanding the effect of particle physicochemical properties on particle-macrophage interactions, it is not known how macrophage functions like cytokine production are affected while presenting active ligands on particles with altered physical properties. Moreover, it is unknown if ligand presentation through an altered particle shape can elicit differential macrophage cytokine responses and if responses are ligand dependent. Therefore, we investigated the influence of geometric particle presentation of diverse ligands, bovine serum albumin, immunoglobulin-G and ovalbumin, on macrophage inflammatory cytokine response. Our results indicate that for similar ligand densities, ligand presentation on rods enhanced production of inflammatory cytokine tumor necrosis factor-alpha (TNF-α) compared to spheres regardless of the nature of the ligand and its cellular receptor. Surprisingly, TNF-α responses were affected by ligand density in a shape-dependent manner and did not correlate to total particle-macrophage association. This study demonstrates the ability of geometric manipulation of particle ligands to alter macrophage cytokine response irrespective of the nature of the ligand.

## Introduction

Nano- and micro particles have been developed to tailor immune responses including enhancement of vaccine responses to pathogens, drug delivery to inflamed cells, and modulation of inflammation in antigen-specific or non-specific manners [1]. New strategies are being explored to control immune functions and particle properties play a key role in programming cellular responses. Particle size [2–4], shape [5–7], surface chemistry [8,9], ligand density [3,10], and surface topography [11] are crucial in governing cellular responses in a wide range of cells. Immune cells, in particular, are quite sensitive to particle properties [5,12–14], which can determine the type of immune response [4,7].

The importance of particle surface charge was highlighted in inflammatory disease models including colitis, peritonitis, and myocardial infarction [15]. Negatively charged spherical particles without any specific antigen were engulfed by inflammatory monocytes, which migrated to the spleen, resulting in apoptosis and dampening of inflammation. Particle shape can also alter immune cell responses to particles. Thiolated poly-(methacrylic acid) (PMA_SH_) rod shaped capsules stimulated macrophages in a shape dependent manner *in vitro*, leading to increased tumor necrosis factor-alpha (TNF-α) production over spherical capsules [13].

These examples demonstrate the ability to tune the cellular and inflammatory outcomes of particles without specific biological ligands. While this understanding is crucial, many particles display functional biomolecules on their surface so there is also a need to understand how particle properties impact presentation of ligands on their surface. Surface presentation of biological ligands that bind cell surface receptors is critical in controlling both the innate [3] and adaptive immune responses [10] to particles, while presentation of synthetic molecules like polyethylene glycol (PEG) can delay innate responses [16]. There is evidence that the cellular response to a biological ligand or synthetic molecule functionalized on a particle surface is altered by the particle shape. West Nile virus antigens coated on spherical and cubic gold nanoparticles induced production of multiple cytokines, including TNF-α, in dendritic cells, while the same antigens on gold nano-rods did not [17]. Conversely, mannose functionalized poly(DL-lactide)-b-poly-(acrylic acid) long cylindrical nanoparticles induced greater IL-6 inflammatory cytokine responses in macrophages than spherical or short cylindrical nanoparticles [18]. In another study, ovalbumin (OVA) conjugated spherical or rod shaped polystyrene (PS) particles induced a Th1 or Th2 type immune response, respectively, against OVA *in vivo* [7]. Ellipsoidal poly(lactide-co-glycolide) (PLGA) particles displaying MHC-Ig dimers and anti-CD28 were shown to activate CD8^+^ T cells *in vitro* better than spherical particles [12]. Phosphatidylserine presented on PLGA rods (rounded ends) suppressed effector T cell function in a myelin-specific multiple sclerosis model by promotion of regulatory T cells *in vitro*. These particles ameliorated autoimmune responses *in vivo*, while phosphatidylserine PLGA cylinders (flat ends) did so to a lesser extent [14]. PEG stimulated production of granulocyte macrophage colony-stimulating factor (GM-CSF) in microglial cells when coated on spherical and rod shaped, but not urchin shaped, gold nanoparticles [19].

Though presentation of ligands on shaped particles alters cellular outcomes, there are no clear trends when comparing these reports. It is possible these results are applicable only to the specific ligand and shape studied. Given the diversity of ligand presentation on engineered particles, it has been a challenge to identify design strategies for shapes and ligands that can be implemented to elicit desired immunological responses. Additionally, it is critical to understand if unintended immunological responses occur when ligand functionalized shaped particles interact in an off-target manner with cells like macrophages.

Here, we demonstrate the role of geometric presentation across varied ligands to macrophages with a controlled particle shape system. Macrophages play an important role in innate immunity by clearing microorganisms and foreign particulate matter, and in adaptive immunity through antigen processing and presentation to lymphocytes [20]. We examined the interplay between ligand presentation and particle shape using bovine serum albumin (BSA), immunoglobulin-G (IgG), and OVA immobilized on spherical and rod shaped PS particles to stimulate macrophage TNF-α inflammatory cytokine production. We investigated the influence of ligand density and particle-macrophage association on shape-dependent TNF-α secretion to explain the effects seen.

## Materials and methods

### Materials

Polyvinyl alcohol (PVA, hydrolyzed degree 99+%, Mw = 85–124 kDa) and ethanol were purchased from Sigma-Aldrich; isopropyl alcohol (IPA) from BDH; BSA from Fischer Scientific; OVA from Invivogen. Fluorescent or plain polystyrene carboxyl functionalized PS particles were purchased from Polysciences. The diameters were 3.1 +/- 0.073 μm and 3.001 +/- 0.144 μm, respectively, as measured by the manufacturer. Antibodies were purchased from: anti-BSA IgG (Life Technologies); Rabbit anti-chicken OVA (Bio-Rad); FITC goat-anti-rabbit IgG (BD Life Sciences). Materials were used as received. Ultrapure water was obtained from a Millipore Synergy UV system (18.2 MΩ).

### Cells

J774 murine macrophages (American Type Culture Collection (ATCC)) were grown in Dulbecco’s Modified Eagle’s Medium (DMEM) (ATCC) at 37˚C in a humidified atmosphere containing 5% CO_2_. The media was supplemented with 10% fetal bovine serum (Seradigm) and 1% penicillin/streptomycin (Amresco). The cells were used between passages 4 and 15.

### Fabrication of rod shaped particles

Our previously reported method was used to fabricate rod PS shaped particles [21]. Briefly, 1 ml of 2.6 wt% fluorescent or plain PS spheres was suspended in polyvinyl alcohol (PVA) solution (0.1 g/ml) and the solution was dried to yield a film of thickness of ~65 μm. The film was stretched in a hot oil bath at 120˚C to an aspect ratio of 2.5. The film was cooled and particles extracted from the film by heating in 30% IPA-water solution at 65˚C. Particles were collected by centrifugation at 2500 g using an Allegra X-15R Centrifuge (Beckman Coulter) for 15 minutes and resuspended 7 times to remove all PVA from the particles.

### Ligand functionalization of particles

Plain or fluorescent shaped PS particles (spherical or rod-shaped) were coated with BSA or OVA using 10 times excess of the surface monolayer saturation calculated from the manufacturer’s protocol (S1 File). Particles were incubated with BSA or OVA (1 mg/ml) in phosphate buffered saline (PBS) for two hours and washed. To functionalize with IgG, BSA coated particles were incubated for one hour with rabbit anti-BSA IgG at a concentration of 1 mg/ml and washed. For variable IgG density on particles, a dilution of 1:5 or 1:100 of IgG to BSA was used. For functionalization analysis, OVA coated particles were incubated for one hour with primary rabbit anti-chicken OVA at a mass ratio of 1:1 (OVA:IgG). To confirm the presence of ligands via anti-BSA or anti-OVA IgG, particles were incubated for 30 minutes with FITC goat-anti-rabbit IgG and washed. All wash steps were performed three times in PBS by centrifugation at 2500g for 5 minutes. The washed particles were assessed by flow cytometry (Accuri C6, Beckton Dickenson Biosciences) to confirm ligand functionalization relative to unfunctionalized PS particles as well as particle concentration.

### Cytokine assay

J774 cells were plated for at least 8 hours at 10^5^ cells per well in a 24 well plate. Ligand coated particles were added to cells in supplemented media (10 particles/cell). Cells were incubated with particles for 8 hours. Supernatants were collected and stored at -80°C. TNF-α production was measured in supernatants by ELISA (R&D Systems) per manufacturer’s instructions. The cytokine assay was repeated three times using three different batches of particles.

### Particle association with cells

J774 cells were plated for at least 8 hours at 10^5^ per well in a 24 well plate. BSA, IgG or OVA functionalized fluorescent particles were added to cells in supplemented media (10 particles/cell). After 8 hours, cells were washed with cold PBS three times and scraped from wells. Cell populations were gated based on side and forward scatter of J774 cells without particles. The number of particles per cell was determined by measuring the mean fluorescence intensity (MFI) of the curve and dividing by the average MFI of one particle [22]. The phagocytic assay was performed twice and 10,000 cells were counted in each condition.

### Enhanced green fluorescent protein (eGFP) adsorption to shaped particles

Particles (30,000) were incubated with eGFP (0.05 mg/ml) for 15 minutes at room temperature. The particles were centrifuged at 2500g for 5 minutes and resuspended in PBS. The particles were immediately interrogated on the flow cytometer. All experiments were repeated in triplicate.

### Statistical analysis

One-way or two-way ANOVA was performed followed by Tukey’s post-hoc test for multiple comparisons. p < 0.05 was considered to be statistically significant in all analysis. Statistics were performed using GraphPad Prism6 software.

## Results and discussion

### Ligand functionalization on spherical and rod shaped particles

**Fig 1:**
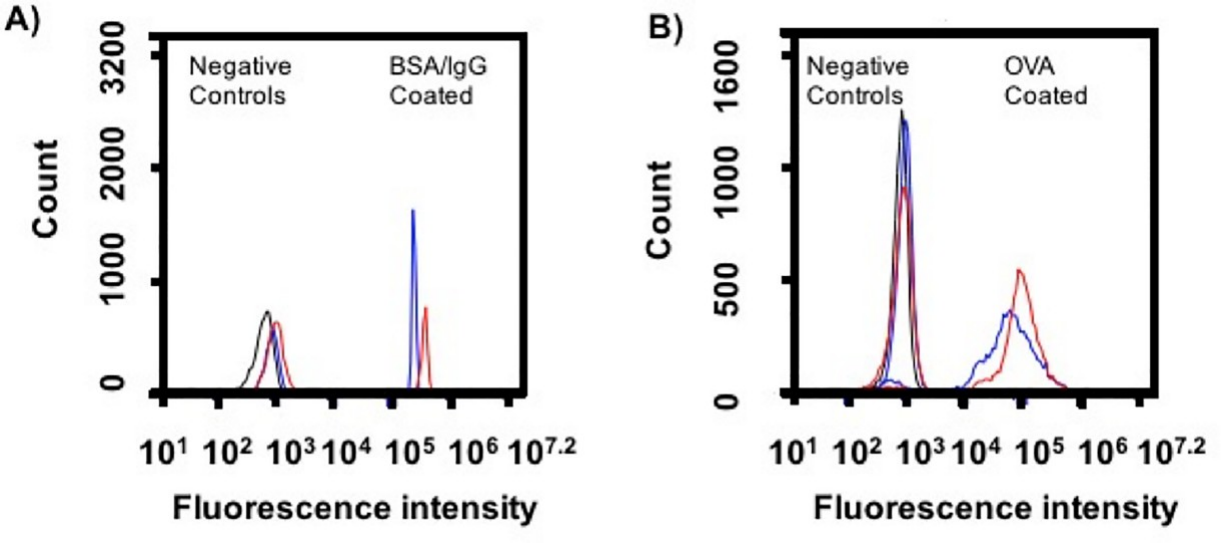
Ligand immobilization on spherical and rod shaped particles. Spherical (blue) and rod (red) shaped particles were adsorbed with ligand (A) BSA and rabbit anti-BSA IgG, or (B) OVA and rabbit anti-OVA IgG, and probed with the same fluorescein isothiocyante (FITC) labeled secondary anti- rabbit IgG to confirm ligand coating. Negative controls (black) were plain particles incubated with BSA (A) or plain particles (B) and FITC labeled secondary anti-rabbit IgG. Representative traces are shown, which were consistent across two independent batches of ligand coated particles.

Rod shaped PS particles (major axis: 12 +/- 0.2 μm and minor axis: 2.5 +/- 0.23 μm, S1 and S2 Figs) were prepared from 3 μm PS spheres by our previously developed method [21]. Particle volume was conserved between spheres and rods, thereby increasing the surface area of rods 1.4 times compared to spheres. We selected diverse ligands, BSA, IgG, and OVA, to present on shaped particles because they interact with macrophages through varied receptors, including scavenger, Fc, and mannose, respectively [23–29]. Ligands were immobilized on particles by passive adsorption. BSA and OVA ligands were directly adsorbed. For IgG immobilization, particles were first coated with BSA, then incubated with anti-BSA IgG to orient the Fc portion of the antibody outwards to Fc receptors on macrophages. Ligand coating was confirmed by incubation with fluorescent secondary antibodies and flow cytometry as seen in Fig 1. Despite the increase in surface area, functionalization of spherical and rod shaped particles resulted in similar ligand immobilization. Further, the area under curve calculations from this data support similar ligand immobilization between shapes (S1 Table).

### Effect of geometric presentation of ligands on macrophage response

**Fig 2:**
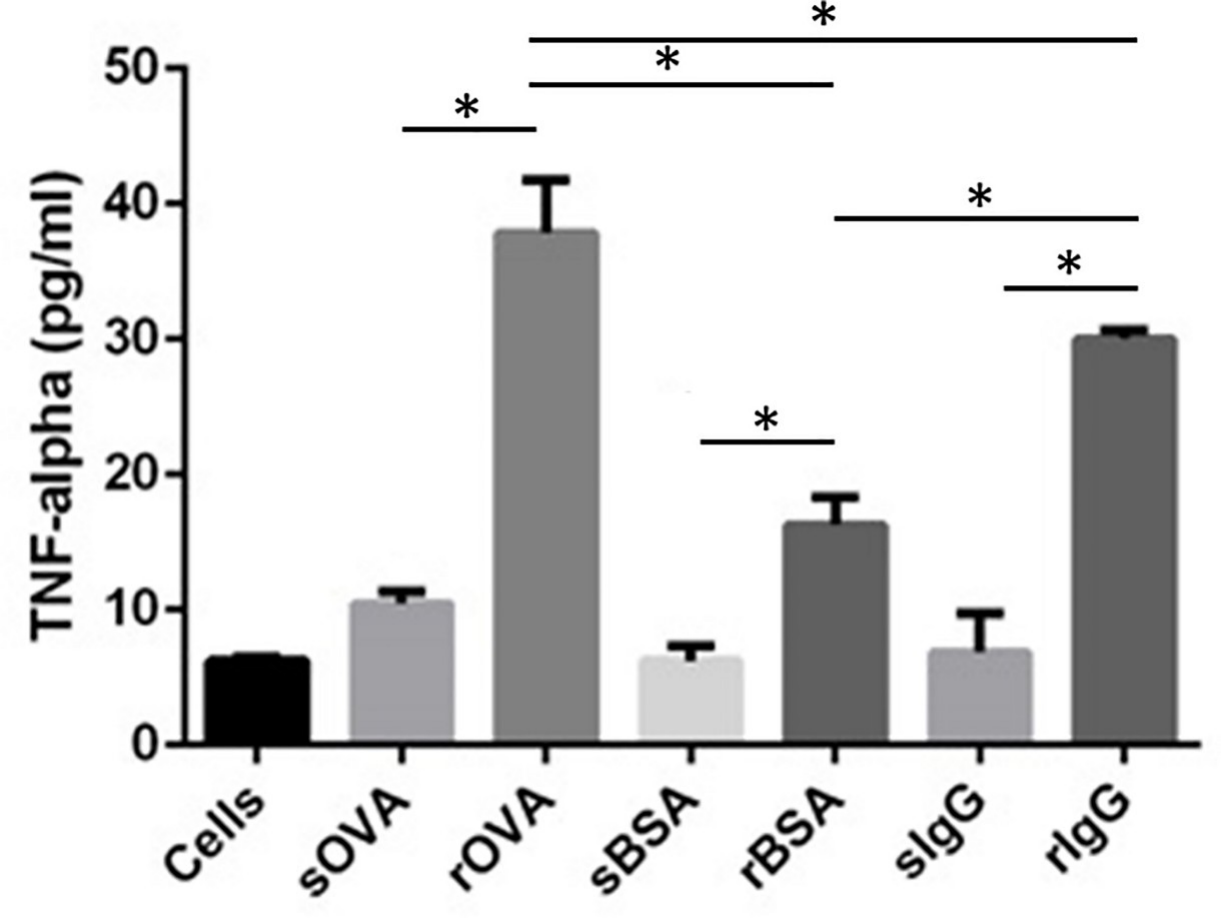
TNF-α production in J774 macrophages in response to ligand presentation on sphere (s) and rod shaped (r) particles. TNF-α values are averages +/- standard deviation of n = 3 and * denotes p<0.05 as determined by one-way ANOVA followed by Tukey’s multiple comparison test.

We determined the effect of geometric presentation of ligands on macrophage inflammatory response by monitoring production of TNF-α, a mediator affected commonly between the scavenger, Fc, and mannose receptor- mediated pathways. TNF-α drives inflammatory responses through activation of the NFκB pathway [30], and rod-shaped PMA_SH_ particles without ligands were reported to induce more macrophage TNF-α secretion than PMA_SH_ spherical particles [13]. We utilized the immortalized murine macrophage cell line J774 for this work as they are more homogenous than primary macrophages and are widely used in evaluating macrophage response to particles *in vitro* [31,32]. We observed significant enhancement of TNF-α secretion by J774 macrophages when ligands of any type were presented on rods compared to spheres (Fig 2). This is similar to increased TNF-α production reported for West Nile antigen coated cubic gold nanoparticles over spherical or rod shapes [17]. While comparisons cannot be drawn, due to differing receptor engagement, particle sizes, and uptake processes, both sets of data indicate that shape and ligand influence the magnitude of inflammatory response. Given the dependence of macrophage TNF-α production on shape of three different particulate-immobilized ligands, it is possible that production of other cytokines, either inflammatory or anti-inflammatory, may be affected by particle shape [13,18]. It is also possible that other macrophage functions, such as oxidative responses or microbial killing, are impacted [33].

Interestingly, functionalization of 3 μm spherical PS particles with ligands IgG and OVA did not enhance TNF-α production by macrophages in comparison to BSA coated spheres. IgG coating on 1 μm PS spheres enhanced TNF-α production by unprimed macrophages compared to BSA coated particles, though the particle dose per cell was 10-fold larger and a different cell line was used [26]. However, in another study, macrophage differential cytokine response between BSA and IgG was lost with increasing size (0.5 to 1 μm) of spherical particles, suggesting the role of size in such signaling [27].

### Relation between IgG ligand density and shape on macrophage response

**Fig 3:**
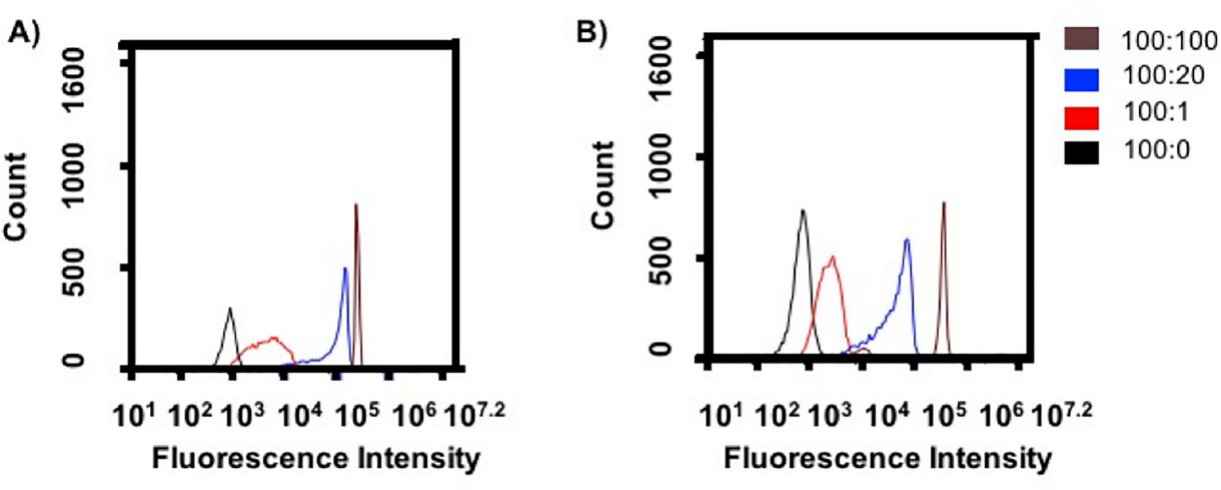
Variable IgG density on spherical and rod shaped particles. Histograms of fluorescence intensity of FITC labeled secondary anti-rabbit IgG added to (A) spherical and (B) rod shaped particles as measured by flow cytometry. Density is presented as the ratio BSA:IgG. Representative traces are shown, which were consistent across two independent batches of ligand coated particles.

Ligand density and particle shape can influence both receptor signaling and uptake by cells [3,10]. For macrophages, a high aspect ratio worm-like shape inhibits phagocytosis [5], while other shapes, like rods, can only be internalized from particular orientations [34]. We, therefore, investigated the role of shape for the same ligand density and the influence of ligand density on a particular shape on TNF-α secretion and total particle association. We chose total particle association with macrophages, which includes attachment and internalization, as both processes influence inflammatory responses during phagocytosis [35]. BSA coated particles were exposed to varying concentrations of anti-BSA IgG antibody to alter the IgG ligand density on each shape. The IgG densities are denoted as a ratio between BSA:IgG and were similar for rods and spheres, as seen in Fig 3.

**Fig 4:**
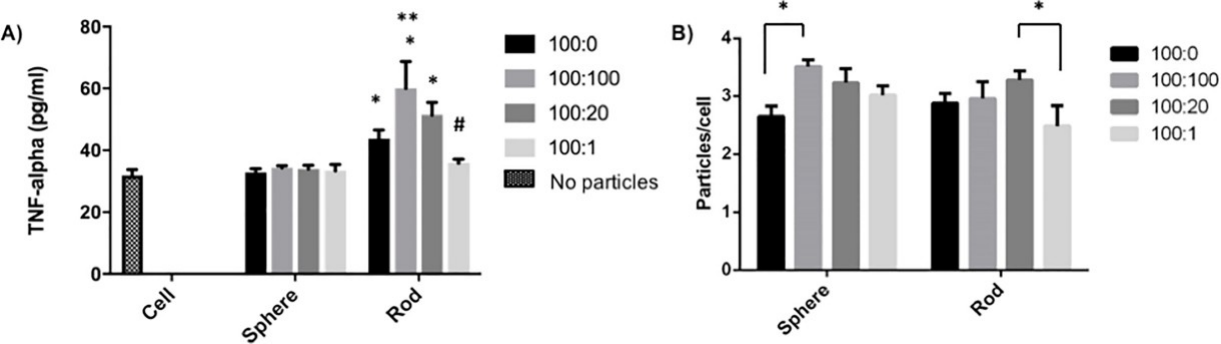
Effect of IgG ligand density and presentation on shaped particles. (A) TNF-α produced by J774 macrophages at each ligand density when presented on spherical or rod shaped particles. (B) Quantitative analysis of total particles associated with macrophages, including attached and internalized, at each ligand density for spheres or rods. Error bars indicate averages +/- standard deviation of n = 3 and * denotes p< 0.05 between corresponding spherical and rod shaped groups. ** denotes p<0.05 between 100:100 and 100:0 rod shaped groups and # denotes p<0.05 between 100:1 and 100:100 or 100:20 rod shaped groups as determined by two-way ANOVA with Tukey’s multiple comparison test.

We measured J774 macrophage TNF-α production for each IgG density on the shapes. Reducing IgG density on spherical particles did not influence TNF-α production by macrophages, as seen in Fig 4A. Similarly, varying IgG density on 1 μm PS spheres did not affect macrophage TNF-α responses [27]. However, reduced IgG density on rod shaped particles correspondingly reduced TNF-α production. Presentation of matched IgG densities on rods produced greater TNF-α production than spheres in all cases except the lowest IgG density, 100:1. In fact, rods functionalized with IgG at 5 times less density (100:20) than spheres (100:100) still elicit a higher TNF-α response, indicating that the shape and not the 1.4x increase in surface area available on rods for ligand immobilization is not responsible for the TNF-α increase.

Using variable IgG functionalized fluorescent spheres and rods, we assessed total particle association, the combination of attached and internalized particles, with J774 macrophages by flow cytometry. Two-way ANOVA was used to determine the influence of both IgG ligand density and its presentation on varying shape on total particle association, as seen in Fig 4B. Maximum IgG functionalization, 100:100, increased macrophage association of spheres compared to non-IgG-functionalized (BSA coated) spheres. Altering IgG density on spherical particles had no influence on total particle association. This is consistent with previous work that reported the role of IgG density on macrophage interactions was negligible for PS spheres 3 μm and larger (3).Conversely, for rods, functionalization with IgG at maximum density (100:100) did not enhance total macrophage association in comparison to BSA coating. Also, reducing IgG ligand density to a ratio of 100:1 on rod shaped particles reduced total particle association compared to the intermediate ligand density ratio of 100:20 but not compared to the maximum ligand density of 100:100. Overall, similar total cellular association was observed for spherical and rod shaped particles at each variable IgG density. This is consistent with our previous study that reported similar total association between IgG coated polyelectrolyte spheres and rods [36]. In the same study, it was established that rods were internalized less than spheres. Macrophage internalization of PMA_SH_ spherical, short and long aspect ratio rod capsules was similar despite differences in TNF-α production [13]. Altogether, this suggests there is no direct correspondence between association or internalization and TNF-α production, and upregulation of TNF-α towards rods over spheres is not due to an increase in cellular association with particles during phagocytosis.

Inflammatory responses initiated by phagocytosis depend on the type of receptor engagement and subsequent particle phagosomal processing [35]. While receptor clustering and subsequent uptake is a hallmark of phagocytosis, it is unknown if receptor clustering varies when particle shape is altered [24]. It is possible shaped particles have a different clustering pattern, which could also depend on particle orientation with respect to the cell, thereby affecting signaling. It has been identified that the concentration of 3’ phosphoinositide stimulated during phagocytosis of rod shaped particles varies with orientation with respect to the cell and reaches high concentrations when approached from the curved side resulting in successful phagocytosis [37,38]. This suggests that signaling during phagocytosis is curvature-dependent, and would be affected by particle shape. Additionally, the role of the ligand and varying ligand density on shaped particles during phagosomal processing in macrophages is unknown. Ligand-receptor engagement and subsequent signaling on spheres could be interpreted as continuous during the binding and/or internalization processes due to their constant curvature. However, since the curvature of rods varies by location and may result in different ligand densities or conformations in different regions of the particle, engagement with receptors and signaling could be discontinuous or exhibit different stages according to the region of the particle interacting with the cell.

Additionally, the role of particle biophysical attributes of ligand presentation in signaling during phagosomal processing in macrophages is unknown. In one study, despite similar acidification of varying sized spherical OVA functionalized protein nanoparticles upon uptake in dendritic cells, medium sized (~ 350 nm) nanoparticles induced larger amounts of TNF-α secretion than small (~ 270 nm) or large nanoparticles (~ 560 nm) [29]. This suggests particle size can alter cytokine responses during surface receptor-mediated pathways. Shape can influence intracellular trafficking in other cell types. PLGA rod shaped microparticles, unlike spheres, oriented tangentially to the nucleus when trafficked in endothelial cells [39]. In another study, disk shaped PS microparticles resided in pre-lysosomal compartments of endothelial cells longer than spherical particles [40]. Taking these size and shape examples together, it is possible that altered phagosomal processing of ligand coated rods influences inflammatory cytokine production.

The conclusion from Figs 2 and 4 that particle shape dominates ligand identity and density in the TNF-α response by macrophages could be driven by evolution of the innate immune response. As key innate immune cells, macrophages play a critical role in recognition and first response to pathogens. While bacterial pathogens display a number of different molecules on their surface, which may be recognized by a variety of different receptors, many bacteria are rod-shaped or non-spherical and shape recognition by macrophages may provide an advantage over, or in addition to surface ligand recognition [41]. While the shapes used in this work are larger than bacteria, the role of shape observed in the cytokine response of macrophages to particles that are similarly and differently sized than bacteria and display different chemistries and ligands [13,17], suggests that particle shape may be universally recognized even though size or chemistry do play a role in the response.

### Effect of particle geometry on adsorbed protein structure

**Fig 5:**
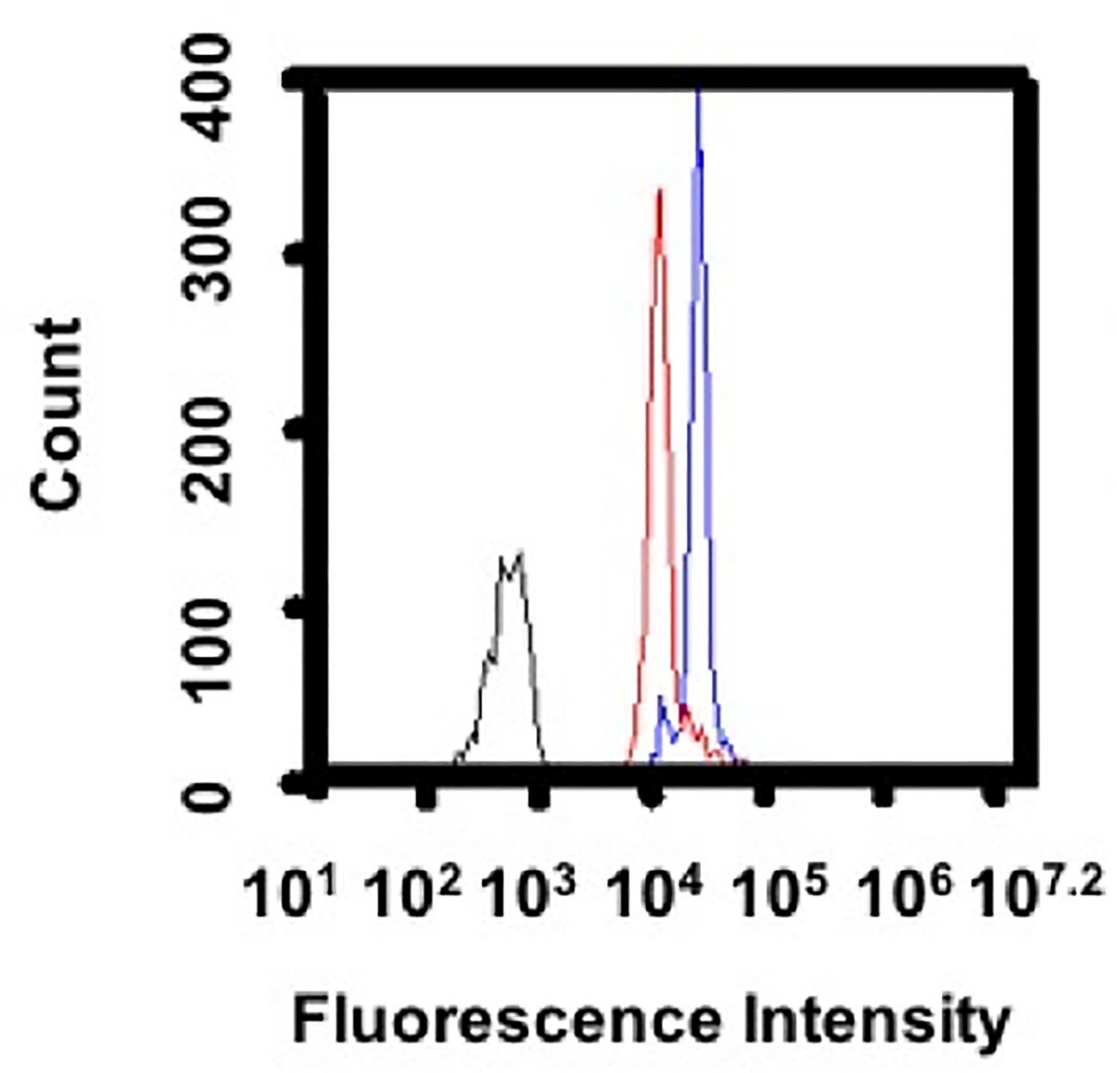
Effect on eGFP structure when adsorbed to shaped particles. Spherical (blue) or rod shaped (red) particles were incubated with eGFP for 15 minutes. Plain particles (black) indicate particle autofluorescence. Representative traces are shown, which were consistent across three independent batches of eGFP coated particles.

Besides altered processing, the structure of adsorbed proteins on the particle surface could influence inflammatory cytokine production [42]. The increase in TNF-α production for BSA and OVA functionalized rods over spheres could be due to a change in the structure of BSA or OVA upon direct adsorption to a rod compared to a sphere. Given the micron-scale size of the particles used, the spheres and sides of the rods have low curvature and likely appear similarly flat to single proteins. However, the curvature at the ends of the rods has been quantified and is significantly higher [34]. These regions of high curvature could induce different orientation or conformation in proteins adsorbing there, and subsequently with cells when binding to these regions. To test the effect of particle shape on protein conformation, spheres and rods were incubated with enhanced green fluorescent protein (eGFP). eGFP fluorescence decreases upon denaturation due to structural changes in its barrel protected chromophore [43,44]. Rods adsorbed with eGFP exhibited lower fluorescence than spheres (Fig 5), even though ligand functionalization levels between shapes were consistent for all ligands (Figs 1 and 3), suggesting eGFP could undergo partial unfolding upon adsorption to rods.

Albumin adsorption to shaped nanoparticles like gold nanorods but not gold nanospheres [45], and cubic [46] or layered silicate nanoparticles [23] leads to denaturation. While the surface chemistry between these shapes is different, it is possible both particle shape and surface chemistry contribute to this effect. PMA_SH_ spherical capsules induce BSA denaturation upon adsorption [47], which could have implications in the study showing PMA_SH_ rod shaped capsules induced more TNF-α than spherical capsules [13]. Curvature variation could also contribute to surface protein denaturation, leading to differential cytokine production. It has been observed that for specific proteins, a surface with greater curvature (small sphere) can promote or hamper the native protein structure in comparison to a ‘flat’ surface (large sphere) [48–50]. Lysozyme adsorbed to 100 nm silica particles experienced a greater loss in secondary structure than 4 nm particles [49]. Similar results were reported for cytochrome-c, human carbonic anhydrase and BSA [48,50,51]. However, fibrinogen adsorption to 165 nm silica particles promoted better structure retention relative to 15 nm particles [51]. Similarly, fibrinogen denatured more on 5 nm than 20 nm poly-acrylic acid coated spherical gold nanoparticles, which led to greater interaction with integrin Mac-1 receptor on macrophages and increased TNF-α production [42]. These studies suggest protein (de)stabilization upon particle adsorption depends not only on particle curvature and chemistry but also on protein properties.

It is possible the structures of BSA, OVA, and IgG are affected by the varying curvature of the rod (major:flat and minor:curved) differently than on the constant, intermediate curvature of the sphere, and these differences contribute to increased inflammatory cytokine secretion for rods. Protein denaturation on particle surfaces can cause alternate receptor engagement and promote inflammatory cytokine production [42,47]. Denaturation of BSA on layered silicate particles reveals cryptic epitopes that promote particle recognition through a specific macrophage scavenger receptor [23] and could explain the difference in TNF-α between spheres and rods.

## Conclusion

We examined the effect of geometric presentation of diverse ligands on macrophage inflammatory TNF-α cytokine production. Our results demonstrate that ligand presentation on rod shaped particles is inflammatory to macrophages when compared to the same ligands on spherical particles. This effect was observed for diverse ligands engaging different macrophage receptors and for varying ligand densities, and differences between types of ligands or ligand densities were amplified on rods relative to spheres. The degree of particle association with cells does not correlate with TNF-α cytokine production by macrophages and is not responsible for the shape effects seen. The ability to program cell-particle interactions through geometric manipulation can be used as a strategy to influence immunological outcomes. Additionally, these results should inform ligand-targeted drug delivery particle design. Whether an inflammatory response to shaped, ligand-coated particles is a desired outcome, as may be in the case of antimicrobial nanoparticle therapies or vaccines, or it is an unwanted negative effect, such as targeted nanoparticles for cardiovascular inflammation, would depend entirely on the application. A shape should be chosen that balances the application goal with any undesired or desired immunological responses caused through the interaction of shaped particles with off-target cells like macrophages.

## Supporting information

S1 File Surface monolayer saturation calculations. (DOCX)

S1 Table Area under the curve (AUC) calculations for particle fluorescence flow cytometry curves in Fig 1 based on trapezoidal rule. Listed is the ratio of rod AUC (red curves in Fig 1) and sphere AUC (blue curves in Fig 1).
(DOCX)

S1 Fig Fluorescent rod shaped polystyrene particles (scale bars 10 μm). (TIFF)

S2 Fig Fluorescent spherical polystyrene particles (scale bars 10 μm). (TIFF)
