# Supplementary material for: Shape of ligand immobilized particles dominates and amplifies the macrophage cytokine response to ligands
Source: PLoS One. 2019 May 17;14(5):e0217022. doi: 10.1371/journal.pone.0217022 (PMC6524819; doi:10.1371/journal.pone.0217022)
Supplement: S1 File — (DOCX) [file pone.0217022.s001.docx]

**S1 File. Surface monolayer saturation calculations.**

The surface monolayer saturation was calculated based on manufacturer’s protocol (Bang’s laboratory Technote 206) using the following formula:

$$S= \frac{6}{\rho D}*C$$

where S = amount of protein required to achieve surface saturation
 (mg protein/g microsphere)

ρ = density of particle (g/cm^3^)

D = diameter (µm)

C = capacity of microsphere surface for a protein
 (mg protein/particle surface)
