# Supplementary material for: Shape of ligand immobilized particles dominates and amplifies the macrophage cytokine response to ligands
Source: PLoS One. 2019 May 17;14(5):e0217022. doi: 10.1371/journal.pone.0217022 (PMC6524819; doi:10.1371/journal.pone.0217022)
Supplement: S1 Table — Listed is the ratio of rod AUC (red curves in Fig 1) and sphere AUC (blue curves in Fig 1). (DOCX) [file pone.0217022.s002.docx]

| *Ligand* | $\frac{\boldsymbol{Rod (red area)}}{\boldsymbol{Sphere (blue area)}}$ |
| --- | --- |
| BSA/IgG | 1.27 |
| OVA | 0.88 |
