# Supplementary figures and images for: Shape of ligand immobilized particles dominates and amplifies the macrophage cytokine response to ligands
Source: PLoS One. 2019 May 17;14(5):e0217022. doi: 10.1371/journal.pone.0217022 (PMC6524819; doi:10.1371/journal.pone.0217022)

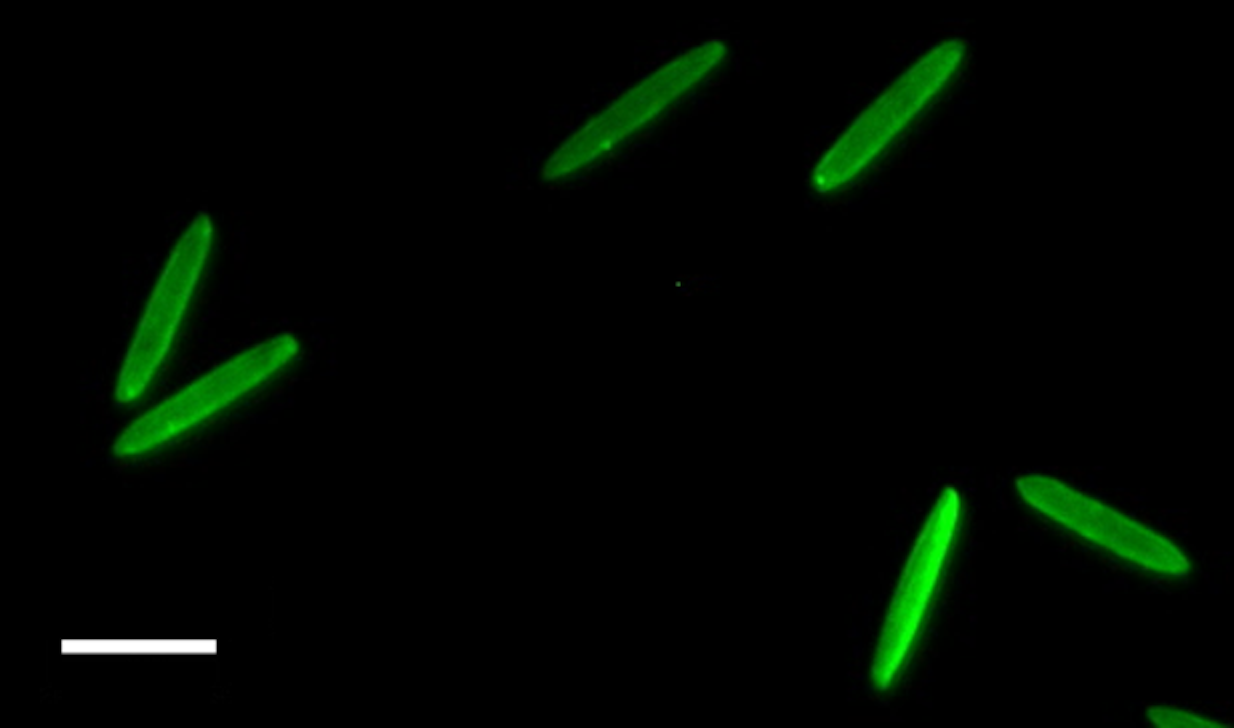

Supplement: S1 Fig — (TIFF) [file pone.0217022.s003.tiff]

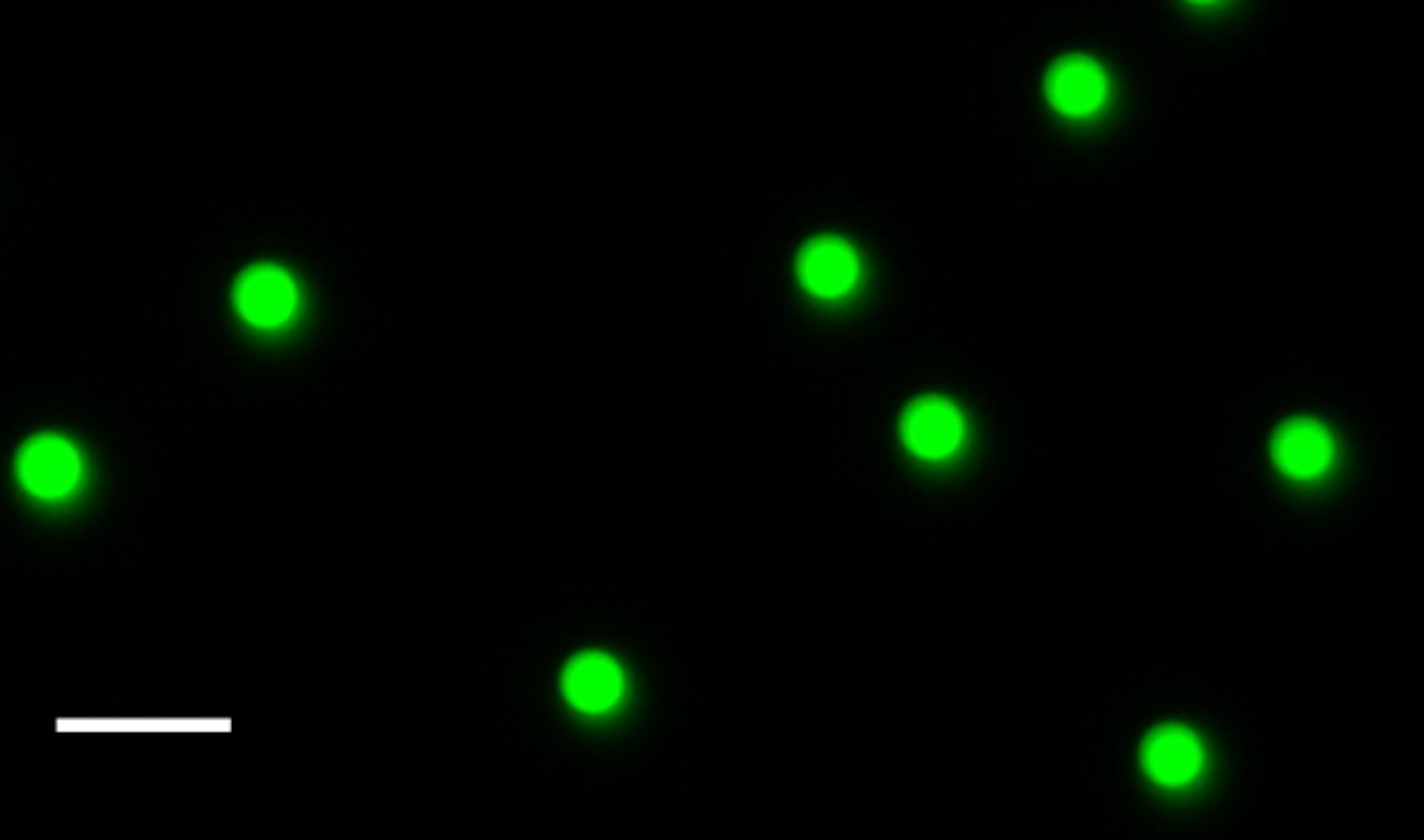

Supplement: S2 Fig — (TIFF) [file pone.0217022.s004.tiff]
